# One-Shot Box-Centric Teaching for Persistent Robotic Sorting-and-Filling with Relative Pose Constraints

**DOI:** 10.3390/s26123703

**Published:** 2026-06-10

**Authors:** Wei Du, Jianhua Wu

**Affiliations:** School of Mechanical Engineering, Shanghai Jiao Tong University, Shanghai 200240, China; 018020210016@alumni.sjtu.edu.cn

**Keywords:** robotic packing task, one-shot learning, programming by demonstration, relative positioning constraints, box-centric template, sorting-and-filling task

## Abstract

Robotic sorting-and-filling tasks in flexible manufacturing require robots to reproduce specified in-box arrangements while adapting to variations in container poses, object availability, sensing conditions, and external interventions. This paper proposes a box-centric one-shot teaching framework for robotic packing tasks with relative pose constraints. In the teaching stage, a human operator demonstrates the desired packing layout only once. The system uses reference-prompted SAM-based contour refinement to extract box and in-box object contours, object categories, quantities, and relative position and orientation constraints. These constraints are then converted from pixel-plane measurements into box-local pose constraints, forming a reusable box-centric packing template that preserves both translational and angular layout information. During execution, the recorded template is transferred to detected box instances with different global poses, and executable pick-and-place commands are generated through a task-level perception-to-command pipeline. A mechanism for continuous assignment and state updates is further introduced to maintain residual target slots, update object-to-slot allocation, and report missing or redundant objects across execution rounds. Single-box template transfer experiments achieved mean placement errors of 7.16 mm and 7.57 mm for two recorded templates, while representative post-execution images further showed that the relative object orientations were visually preserved with respect to the taught template footprints. Multi-box experiments demonstrated that unfinished residual slots could be preserved and completed after scene updates without re-teaching. Additional validation with different container types and object shapes showed the feasibility of extending the framework beyond cube-only cases. Ablation tests under nine exposure settings further showed that SAM refinement improved template-acquisition robustness compared with the previous recognition method. These results verify that the proposed framework enables one-shot template acquisition, box-centric layout transfer, relative pose preservation, and persistent task-level execution for constrained robotic packing tasks.

## 1. Introduction

In modern automated logistics and flexible manufacturing, robotic manipulation has evolved from unstructured bin picking toward structured sorting-and-filling operations where objects must be placed into target containers under strict spatial constraints. Tasks such as kitting, assembly, and order fulfillment therefore require not only reliable singulation and transport, but also accurate in-box placement that conforms to a desired layout. As production continues to shift toward high-mix, low-volume settings, robotic systems must be rapidly deployable, reusable across different containers, and robust to dynamic changes in the workspace.

To address this practical requirement, this article studies a one-shot-taught, box-centric, and dynamically updated sorting-and-filling task. In this setting, a human operator demonstrates the desired arrangement inside a box only once, and the robot must later reproduce the taught layout across multiple target boxes in a changing scene. This formulation introduces three coupled challenges. First, a goal representation defined in a global frame or as a raw image template is sensitive to changes in camera viewpoint and box relocation, and thus does not naturally generalize to different box poses. Second, a demonstrated layout must be transformed into an explicit target-slot representation rather than merely recorded as a raw trajectory, so that the robot can reproduce the in-box arrangement under new container poses. Third, the environment is inherently dynamic: objects may be added, removed, or repositioned during execution, making static open-loop planning insufficient.

Recent studies have improved one-shot teaching and rearrangement transfer from human demonstrations. One-shot visual imitation for multi-step pick-and-place tasks from a single video demonstration has demonstrated the potential for rapid task transfer [1]. Demonstration-based industrial sorting reduces manual programming effort for robotic sorting tasks [2]. One-shot learning from observation transfers task structure from limited human observations [3]. Category-level visual imitation further improves task-oriented grasping and rearrangement by learning visual task representations [4]. In parallel, template-based imitation with symmetry-aware pose handling records reusable geometric relations for symmetric-object manipulation [5]. Off-line classification-oriented programming with feature generalization also reduces programming effort for demonstration-based sorting [6]. However, these methods mainly focus on single object-target relations, trajectory replay under comparatively static assumptions, or offline task reproduction, and they do not directly provide a reusable box-centric layout description for precise multi-slot filling across multiple containers.

At the task-reasoning level, semantics-aware hierarchical decision frameworks use semantic scene encoders and memory to reason over embodied room rearrangement [7]. Point-cloud-based rearrangement methods represent object transformations directly from scene geometry [8]. Scene-prediction-based rearrangement evaluates candidate pick-and-place actions by predicting future point-cloud states [9]. Persistent-environment rearrangement studies also show the importance of considering future task states when executing long-horizon object rearrangement [10]. Nevertheless, these approaches generally target generic rearrangement, scene restoration, or tabletop object reordering, and they often assume that the desired target configuration has already been specified in an execution-ready form. Consequently, the joint problem of one-shot-taught box layouts, reusable container-centric goal representation, and continuous assignment and state updates in dynamic multi-box filling remains insufficiently addressed.

**Figure 1:**
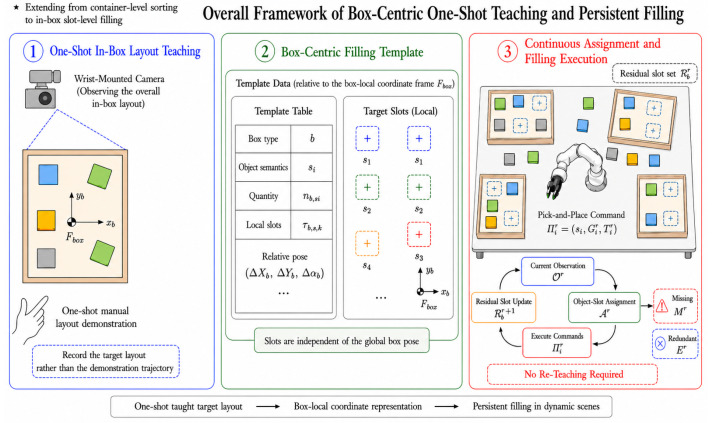
Overall framework of the box-centric one-shot teaching and persistent filling method.

To overcome these limitations, we propose a box-centric, one-shot teaching framework for persistent robotic sorting-and-filling tasks. As illustrated in Figure 1, the proposed framework extends container-level sorting to in-box slot-level filling by integrating one-shot layout teaching, box-centric template construction, and continuous assignment-based execution. In the teaching stage, a human operator manually arranges the required objects inside a target box only once, while a wrist-mounted camera observes the demonstrated in-box layout. The system then extracts the box contour, in-box object contours, object categories, quantities, and relative pose constraints from the demonstrated layout. Instead of recording the demonstrated trajectory, the extracted information is organized as a reusable box-centric filling template, in which object semantics, required quantities, box-local target slots, and relative poses are defined with respect to the local coordinate frame of the box. This representation makes the taught layout independent of the demonstrated box’s global pose and allows the same template to be transferred to newly detected box instances. During execution, the system observes the current workspace, assigns available objects to valid residual slots, generates executable pick-and-place commands, and updates the residual slot state after each round. As a result, missing and redundant objects can be reported, and the filling task can continue after scene changes without re-teaching the layout.

Based on the above framework, the main contributions of this work are summarized as follows.

1. A box-centric, one-shot teaching representation that encodes the taught in-box arrangement as constrained relative object poses, thereby improving invariance to the global location and orientation of the target container.
2. A semantic-grouped template extraction and transfer mechanism that converts the demonstrated layout into object categories, quantities, and box-local target slots, enabling the same taught arrangement to be reused for new box instances.
3. A continuous assignment and state update mechanism that maintains object instances, target-slot occupancy, and residual task allocation across successive execution rounds, enabling persistent sorting-and-filling under dynamic external interventions.

## 2. Related Work

The problem addressed in this paper is situated at the intersection of demonstration-based task specification, spatial arrangement representation, and long-horizon rearrangement planning. To better position the proposed framework, the related literature is reviewed from two complementary perspectives, namely, one-shot teaching and goal representation, and rearrangement planning and execution in dynamic scenes.

### 2.1. One-Shot Teaching and Goal Representation

Robot learning from demonstration has been widely studied as a way to transfer task knowledge from human examples to robots [11]. Recent surveys further summarize how demonstration-based methods encode, generalize, and reproduce robot skills under different task settings [12].

One line of research studies how rearrangement goals are specified for the robot. High-level goal descriptions can be expressed through formal spatial specifications [13], while interactive frameworks can teach novel visual concepts and task structures through language-annotated scene graphs and a single demonstration [14]. More direct demonstration paradigms instead learn desired tasks from human observations. One-shot visual imitation for multi-step pick-and-place tasks [1] learns task execution from a single video demonstration by combining visual perception, trajectory segmentation, and skill representation. Meta-learning-based visual imitation has also been studied for learning new manipulation tasks from a single visual demonstration [15]. One-shot imitation learning has further been formulated as task generalization from a single demonstration to new instances of the same task [16]. Keyframe-based learning from demonstration provides another compact way to represent human-taught task states [17]. Video-based representation learning can support imitation from observations by learning task-relevant representations from videos [18]. Recent work further analyzes one-shot imitation learning from the perspective of object pose estimation and spatial generalization [19]. Template-based imitation learning for manipulating symmetric objects [5] records inter-object frame relations as reusable templates while resolving symmetry-induced pose ambiguity through symmetry detection and template matching. One-shot learning from observation for grasp-manipulation-release household operations [3] also transfers task structure from limited demonstrations. These methods substantially reduce programming effort, but their goal representations are often tied to specific object relations, camera views, or predefined task abstractions.

A second line of research examines the type of task knowledge that should be extracted from demonstrations or contextual evidence. Template-based relational encoding [5] indicates that storing geometric relations between manipulated entities can support task reproduction more directly than purely trajectory-level replay. Object placement has also been studied from the viewpoint of stability, semantic preference, and object–area compatibility [20]. Language-conditioned manipulation methods combine semantic and spatial pathways to support tabletop manipulation from language instructions [21]. Language-guided semantic rearrangement further learns spatial structures for arranging novel objects according to structured language commands [22]. In parallel, personalized rearrangement methods infer user-specific preferences from prior and current scene contexts [23]. Other works focus on structured spatial abstractions, including symbolic scene identification for spatial arrangement [24] and interaction-centric geometric transfer from a single demonstration [25]. Category-level visual imitation methods further learn richer feature representations for task-oriented grasping and rearrangement [4]. Collectively, these studies capture useful semantic, relational, or geometric priors, but most of them target general placement preferences, qualitative relations, category-level transfer, or pairwise object relations rather than a reusable container-anchored template for exact in-box filling.

### 2.2. Rearrangement Planning and Execution in Dynamic Scenes

Once the goal has been specified, the system must decide how to realize the rearrangement under geometric and temporal constraints. Explicit state-based planning remains a major paradigm in this area. In demonstration-oriented sorting programming, the off-line programming framework based on human demonstration [6] records sorting trajectories, object-to-box correspondences, and key turning features with a single depth camera, and then generalizes the demonstrated path to new positions of objects and boxes.

At a broader planning level, semantics-aware hierarchical decision frameworks use semantic scene encoders and memory to reason over embodied room rearrangement [7]. Visually guided rearrangement has also been formulated as a search problem over object-moving sequences from visual observations [26]. Similarly, SPOT plans directly on point-cloud object transformations in continuous action spaces [8]. Flow-based rearrangement methods estimate object transformations between initial and goal RGB-D scenes for object rearrangement [27]. Multi-object sorting and rearrangement have also been studied using Monte Carlo tree search in planar nonprehensile tasks [28]. These methods are powerful for structured long-horizon reasoning, yet they typically presuppose that the target arrangement is already available in a suitable symbolic, geometric, or point-cloud form.

An alternative direction reduces dependence on explicit symbolic reasoning at the scene level by learning action selection more directly from observation. Scene-prediction-based rearrangement learns to predict future point-cloud scenes to evaluate candidate pick-and-place actions [9]. Compared with the offline sorting-oriented programming approach in [6], which preserves interpretable target correspondences and key-path features, these methods reduce the need for hand-crafted task abstractions but make it more difficult to maintain explicit box-level templates, slot-occupancy states, and object-to-slot assignment states throughout persistent execution.

For dynamic and persistent environments, planning must account for the effect of current actions on future tasks. Persistent-environment rearrangement methods highlight the importance of considering downstream task states when making current rearrangement decisions [10]. Even demonstration-based sorting frameworks that can generalize taught trajectories to new object and box placements [6] generally remain trajectory-centric and off-line in nature, rather than continuously updating box occupancy, object availability, and assignment decisions while external additions and removals occur during execution. Accordingly, fewer works study a setting in which the desired final state itself is taught once by a human, represented relative to a movable box, and then continuously maintained across execution rounds as objects and containers change during the task.

In contrast to the above literature, the present work couples one-shot box-centric teaching with explicit residual-slot maintenance. The proposed framework is therefore designed not only to infer or plan a rearrangement, but also to preserve a reusable in-box layout specification and to keep that specification executable under dynamic scene changes.

## 3. Methodology

### 3.1. System Layout and Notation

**Figure 2:**
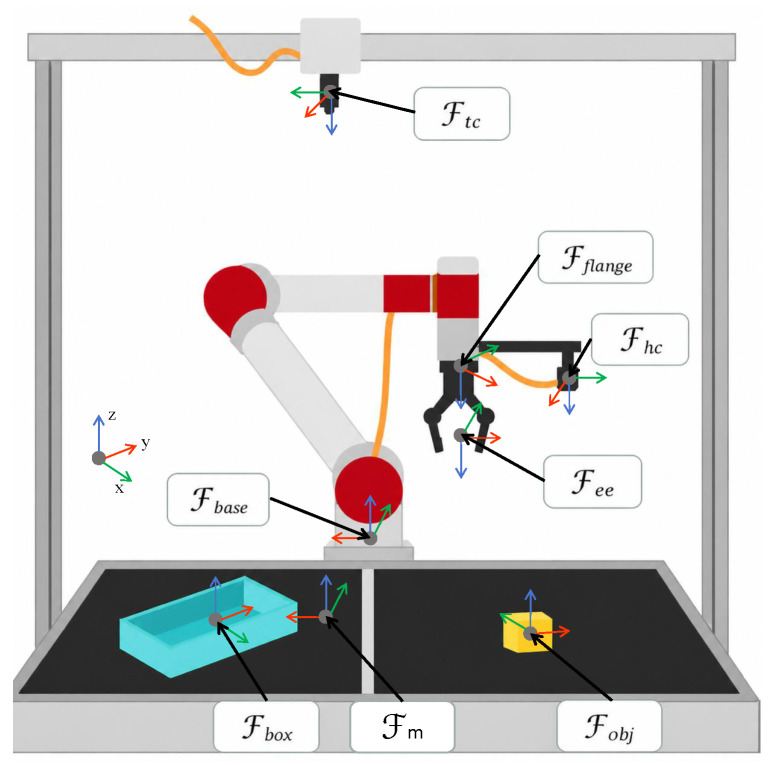
Overall framework of the proposed box-centric one-shot teaching and persistent filling method.

As shown in Figure 2, the system consists of a robot manipulator mounted beside the workstation, an overhead RGB-D camera, and a wrist-mounted RGB-D camera installed near the robot gripper. The whole workspace is covered by the overhead camera, while the wrist-mounted camera is used for local observation during template acquisition and pose extraction. The containers and the objects to be packed are placed in the workspace.

The basic sensing and recognition module used in this work follows our previous feature-reserved teaching framework for pick-and-place systems [29]. Specifically, this module provides the initial color-based object and box recognition, contour extraction, semantic category identification, and reference pixel point acquisition. In the present framework, it is used as the front-end perception component for obtaining coarse box/object information and reference points. The subsequent SAM-based refinement and box-centric template construction are then introduced to improve contour accuracy and convert the detected layout into reusable relative pose constraints.

The coordinate frames used in this work are also shown in Figure 2. Specifically, $F_{base}$, $F_{flange}$, $F_{ee}$, $F_{tc}$, $F_{hc}$, $F_{obj}$, and $F_{box}$ denote the robot base frame, the robot end-flange frame, the gripper end-effector frame, the overhead-camera frame, the hand-camera frame, the object frame, and the box frame, respectively. The robot base frame $F_{base}$ is also used as the world frame.

The pose of each object or box is represented by three translational components and a yaw angle about the *z*-axis. The notation Pif∈R3 denotes the position of item *i* expressed in the frame $F_{f}$, and αif denotes its yaw angle in the same frame. When the pose is estimated from a camera observation, the superscript cam is used, such as Picamf and αicamf, where cam∈{tc,hc} denotes the overhead camera or the wrist-mounted camera. The homogeneous form of Pif is denoted by P˜if∈R4. The homogeneous transformation from frame $F_{b}$ to frame $F_{a}$ is denoted by Tab. The symbol Δ is used to represent relative quantities between an object and a box, including pixel-plane displacements, metric displacements, and relative orientation angles, which are defined when they are first introduced.

Since observations from the overhead and wrist-mounted cameras are both expressed in their own camera frames, the estimated poses are converted to the base frame for Cartesian path planning. For an item observed by the overhead camera, its position and yaw angle in the base frame are calculated as

(1) P˜itcbase=Ttcbase·P˜itctc

(2) αitcbase=αtcbase+αitctc

For an item observed by the wrist-mounted camera, its position is transformed to the base frame through the hand-eye calibration chain, while its yaw angle is refined by adding the wrist-camera yaw correction to the overhead-camera coarse estimate. This additive yaw refinement is valid because the overhead camera and the wrist-mounted camera are configured with parallel imaging planes during local observation.

(3) P˜ihcbase=Tflangebase·Thcflange·P˜ihchc

(4) αihcbase=αitcbase+αihchc=αtcbase+αitctc+αihchc

It should be noted that this work does not aim to design a new low-level robot controller or visual-servoing law. Instead, the proposed framework provides a task-level execution interface between perception, teaching, assignment, and robot motion. After the grasping pose $G_{i}^{r}$ and the target placement pose $T_{i}^{r}$ are generated, the corresponding pick-and-place command $\Pi_{i}^{r}$ is sent to the robot controller and executed through the built-in Cartesian motion planning and position-control functions of the manipulator. Therefore, the control-related contribution of this work lies in perception-guided command generation, task-state feedback, and residual-slot maintenance, rather than in joint-level servo control or force-control design.

### 3.2. Problem Statement

In a typical sorting-and-filling task, multiple objects with specified semantic categories and quantities must be placed into a target container according to the desired in-box layout. Different box types may correspond to different object requirements and spatial arrangements. Therefore, manually programming each packing order through hard-coding, pendant teaching, or drag teaching is inefficient in high-mix and low-volume production scenarios.

In this work, the operator only needs to provide one packing demonstration for each box type. After the demonstration, the system observes the demonstrated box and extracts the packing information, including the box type, the semantic categories and quantities of the objects, and the relative pose constraints of the objects with respect to the box frame. Since the demonstrated layout is observed by the wrist-mounted camera, the object and box poses obtained in $F_{hc}$ are first transformed into the base frame according to Equations (3) and (4), resulting in the base-frame pose representation (Pihcbase,αihcbase). Based on this transformed pose representation and the refined pixel-plane relative constraints, the demonstrated in-box arrangement is further converted into box-centric target slots. The categories, quantities, and target slots of the extracted objects are organized into a reusable packing template $G_{b}$, rather than as a trajectory that can be directly replayed.

The execution problem is then formulated as a continuous state maintenance process. For each detected box *b*, the system maintains a residual slot set $R_{b}^{r}$ in the *r*-th execution round to describe the target slots that have not yet been filled. In each round, the overhead camera observes the currently available objects outside the box regions and forms the scene observation set $O^{r}$. Based on $O^{r}$, the current box poses, and the residual slot sets $\left\{R_{b}^{r}\right\}$, the system determines the object-to-box assignment set $A^{r}$ and generates executable pick-and-place commands $\Pi_{i}^{r}$ for the assigned objects.

After each execution round, the slots corresponding to successfully executed commands are removed from the residual slot sets. The missing set $M^{r}$ records the categories and quantities of objects that are still required by each box, while the redundant object set $E^{r}$ records the observed objects that are not assigned to any box in the current round. These variables constitute the task state maintained by the continuous assignment and state update mechanism, which is formally defined in the following subsections.

### 3.3. Packing Relations and Position Constraints Recognition

During one-shot teaching, the demonstration box is observed by the wrist-mounted camera to capture the in-box arrangement with sufficient local detail. The basic perception module identifies the box type and its pixel-level position. Based on the information pre-stored in the established object library, the module further recognizes the semantic categories and quantities of the objects inside the box, as well as the predefined indicator pixel points of each object instance.

#### 3.3.1. Pixel-Plane Position Constraints Recognition

Considering that the reference pixel point provided by the basic perception module is obtained by averaging the pixel coordinates within a contour extracted using an HSV threshold-based mask, the extracted reference point is susceptible to illumination variations, shadows, and color interference from surrounding objects. As a result, this reference point may deviate from the true planar geometric center of the object. To address this issue, the reference pixel point is used as a prompt point for the Segment Anything Model (SAM; Meta AI, Meta Platforms, Inc., Menlo Park, CA, USA) [30], and the images captured by the wrist-mounted camera are re-segmented to obtain refined object contours. As a prompt-based visual foundation model, SAM supports flexible image segmentation using prompts such as points, bounding boxes, and masks, and can be applied to both global scene segmentation and local object segmentation tasks. Its zero-shot generalization capability and segmentation accuracy reduce the need for task-specific training data, thereby improving the adaptability of the perception module.

Algorithm 1 illustrates how the basic perception module is combined with SAM to obtain the relative constraints in the pixel plane. The reference pixel point of the *i*-th object is denoted by $\left(u_{i}^{ref},v_{i}^{ref}\right)$, and its semantic label is denoted by $s_{i}$. The refined pixel position and orientation of the object and the box are represented by $\left(u_{i},v_{i},\alpha_{i}\right)$ and $\left(u_{b},v_{b},\alpha_{b}\right)$, respectively. The goal of the algorithm is to obtain $\left(\left(\Delta u_{b,i},\Delta v_{b,i}\right),\Delta \alpha_{b,i},s_{i}\right)$ for each object in the demonstrated box *b*. The conversion from these pixel-plane constraints to box-local metric pose constraints is described in the following subsection.
**Algorithm 1** SAM-based Pixel-Plane Box-Object Segmentation and Relative Constraint Recognition Algorithm  1:**Input:** Original image *I*, object anchor points $A=\{a_{1},a_{2},\ldots ,a_{N}\}$ with $a_{i}=\left(u_{i}^{\mathrm{ref}},v_{i}^{\mathrm{ref}}\right)$, semantic labels $S=\{s_{1},s_{2},\ldots ,s_{N}\}$, pre-trained SAM  2:**Output:** Pixel-plane relative constraint set $\Delta P={\left\{\left(\left(\Delta u_{b,i},\Delta v_{b,i}\right),\Delta \alpha_{b,i},s_{i}\right)\right\}}_{i=1}^{N}$ with respect to box *b*  3:Perform anchor-free SAM segmentation on *I* to obtain all target masks $M_{\mathrm{all}}$  4:Calculate the pixel area of each mask and extract the maximum-area mask as the box mask: $M_{b}=\arg\max_{m\in M_{\mathrm{all}}}\mathrm{Area}\left(m\right)$  5:Obtain the minimum rotated bounding rectangle $Rect_{b}$  6:Extract the four corners of $Rect_{b}$: $r_{b,1}$, $r_{b,2}$, $r_{b,3}$, $r_{b,4}$, and calculate the box center $\left(u_{b},v_{b}\right)$:
$$u_{b}=\frac{1}{4}\cdot \sum_{k=1}^{4}r_{b,k}.u,\;v_{b}=\frac{1}{4}\cdot \sum_{k=1}^{4}r_{b,k}.v$$
  7:Extract the unified rotation angle of $Rect_{b}$ as the box angle: $\alpha_{b}=\mathrm{Angle}\left(Rect_{b}\right)$  8:Initialize pixel-plane relative constraint set ΔP=⌀  9:**for** i=1 *N* **do**10:    Take the *i*-th anchor $a_{i}$ and semantic label $s_{i}$, and perform anchor-based SAM segmentation on *I* to obtain object mask $M_{i}$11:    Extract the edge contour points of $M_{i}$ and fit a minimum rotated bounding rectangle $Rect_{i}$12:    Extract the four corners of $Rect_{i}$: $r_{i,1}$, $r_{i,2}$, $r_{i,3}$, $r_{i,4}$, and calculate the object center $\left(u_{i},v_{i}\right)$$$u_{i}=\frac{1}{4}\cdot \sum_{k=1}^{4}r_{i,k}.u,\;v_{i}=\frac{1}{4}\cdot \sum_{k=1}^{4}r_{i,k}.v$$
13:    Extract the unified rotation angle of $Rect_{i}$ as the object angle: $\alpha_{i}=\mathrm{Angle}\left(Rect_{i}\right)$14:    Calculate the relative pixel displacements in the image plane:
$$\Delta u_{b,i}=u_{i}-u_{b},\;\Delta v_{b,i}=v_{i}-v_{b}$$
15:    Calculate the relative rotation angle in the pixel plane and normalize it to [−π/2,π/2]$\Delta \alpha_{b,i}=\mathrm{mod}\left(\alpha_{i}-\alpha_{b}+\pi /2,\pi \right)-\pi /2$
16:    $\Delta P=\Delta P\cup \{\left(\left(\Delta u_{b,i},\Delta v_{b,i}\right),\Delta \alpha_{b,i},s_{i}\right)\}$17:**end for**18:**return** ΔP

#### 3.3.2. Box-Local Pose Constraint Calculation

This section converts the pixel-plane relative constraints into box-local pose constraints. Here, $\Delta u_{b,i}$ and $\Delta v_{b,i}$ denote the displacement components in the pixel plane from the center of box *b* to the center of object *i*, while $\Delta \alpha_{b,i}$ denotes the relative orientation between object *i* and box *b* in the pixel plane. After inverse perspective projection, $\Delta X_{b,i}^{m}$ and $\Delta Y_{b,i}^{m}$ denote the corresponding metric displacement components expressed in the intermediate frame $F_{m}$. After planar coordinate transformation, $\Delta X_{b,i}^{b}$, $\Delta Y_{b,i}^{b}$, and $\Delta \alpha_{b,i}^{b}$ denote the local pose constraints of object *i* with respect to box *b* expressed in the box frame $F_{b}$.

**Figure 3:**
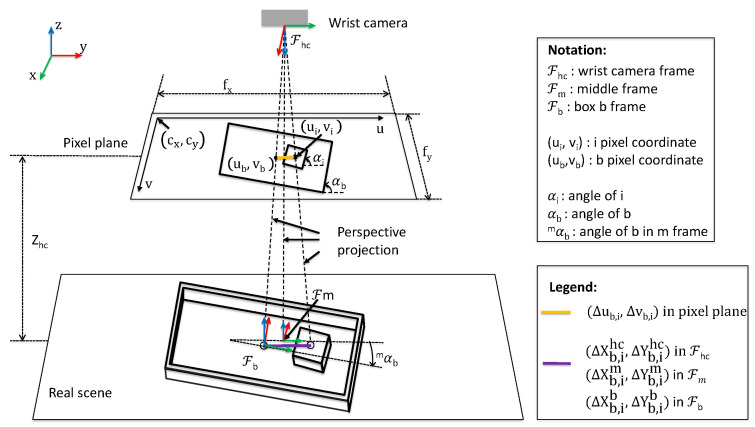
Geometric relationship between pixel-plane constraints and box-local pose constraints.

As shown in Figure 3, the conversion consists of two geometric steps. First, the pixel-plane displacement $\left(\Delta u_{b,i},\Delta v_{b,i}\right)$ is converted into a metric displacement using the wrist-camera geometry, the perpendicular observation distance $Z_{hc}$, and the camera focal parameters $f_{x}$ and $f_{y}$. Second, the metric displacement is re-expressed from the intermediate frame $F_{m}$ to the box frame $F_{b}$ by a planar rotation determined by the box orientation. This geometric interpretation corresponds to the following inverse perspective projection and frame-rotation equations.

An intermediate reference frame $F_{m}$ is introduced to perform this conversion, as shown in Figure 3. The origin of $F_{m}$ is set as the perpendicular projection of the optical center of the wrist-mounted camera onto the desktop during observation. Its *x*-axis is in the same direction as the camera *x*-axis, its *y*-axis is opposite to the camera *y*-axis, and its *z*-axis is perpendicular to the desktop and points outward. During local observation, the wrist-mounted camera is adjusted to observe the desktop approximately along the surface normal, and the box and object centers are assumed to lie on the same support plane. The perpendicular observation distance $Z_{hc}$ denotes the distance from the optical center of the wrist-mounted camera to this support plane. It is calculated from the extrinsic parameters of the wrist-mounted camera and the current pose of the robot end effector, and remains constant throughout the observation process. Based on the inverse perspective projection principle and the spatial relationship between $F_{m}$ and $F_{hc}$, the coordinate differences of the object relative to the box in $F_{m}$ are obtained from $\left(\Delta u_{b,i},\Delta v_{b,i}\right)$. Here, $f_{x}$ and $f_{y}$ denote the pixel-scale focal lengths of the camera along the *x*- and *y*-axes, respectively. For the demonstrated box *b*, $\Delta X_{b,i}^{m}$ and $\Delta Y_{b,i}^{m}$ are computed as follows:

(5) ΔXb,im=ΔXb,ihc=Δub,i·ZhcfxΔYb,im=−ΔYb,ihc=−Δvb,i·Zhcfyαbm=−αbhc

The box angle extracted on the wrist-camera image plane is denoted as αbhc, corresponding to $\alpha_{b}$ in Algorithm 1. The negative signs in the *y*-direction and in the angular term result from the opposite directions of the wrist-camera image *v*-axis and the *y*-axis of $F_{m}$.

Since $F_{m}$ and $F_{b}$ differ only by a planar translation and a rotation about the *z*-axis, the translation term is canceled for the object-to-box displacement. Thus, the coordinate differences expressed in $F_{b}$ can be obtained from those in $F_{m}$ through a planar rotation transformation:

(6) ΔXb,ibΔYb,ib=cos(αbm)sin(αbm)−sin(αbm)cos(αbm)·ΔXb,imΔYb,im

Combining Equations (5) and (6), the coordinate and angular deviations of the object relative to the box in $F_{b}$ observed by the wrist-mounted camera can be expressed as

(7) ΔXb,ibΔYb,ibΔαb,ib=Δub,i·Zhcfx·cos(αbhc)+Δvb,i·Zhcfy·sin(αbhc)Δub,i·Zhcfx·sin(αbhc)−Δvb,i·Zhcfy·cos(αbhc)−Δαb,i

The negative sign in $\Delta \alpha_{b,i}^{b}$ is consistent with the angular relationship between the wrist-camera image plane and $F_{m}$. The set ΔB denotes the local pose constraints in the box obtained by updating the coordinate and angular information in ΔP. Each element of ΔB is represented as $\left(\Delta X_{b,i}^{b},\Delta Y_{b,i}^{b},\Delta \alpha_{b,i}^{b},s_{i}\right)$.

#### 3.3.3. Semantic-Grouped Object Statistics

After the box-local pose constraint set ΔB is obtained, Algorithm 2 groups the object instances according to their semantic labels and constructs the box-centric packing template for box type *b*. Each element in ΔB is represented as $\left(\Delta X_{b,i}^{b},\Delta Y_{b,i}^{b},\Delta \alpha_{b,i}^{b},s_{i}\right)$, where $s_{i}$ is the semantic label of object instance *i*. During grouping, $s_{i}$ is used to determine the corresponding semantic category *s*, and a target slot instance is then created for this object.
**Algorithm 2** Semantic-Based Grouping and Box-Centric Template Construction  1:**Input:** Box-local pose constraint set ΔB from Equation (7)  2:     and box type label *b*  3:**Output:** Box-centric packing template $G_{b}$ for box type *b*  4:Initialize semantic category set $S_{b}\leftarrow ⌀$  5:Initialize box-centric packing template $G_{b}\leftarrow ⌀$  6:**for** each element $(\Delta X_{b,i}^{b},\Delta Y_{b,i}^{b},\Delta \alpha_{b,i}^{b},s_{i})\in \Delta B$ **do**  7:    Set the semantic category $s\leftarrow s_{i}$  8:    **if** $s\notin S_{b}$ **then**  9:        Update $S_{b}\leftarrow S_{b}\cup \left\{s\right\}$10:        Initialize the required object number $n_{b,s}\leftarrow 0$11:    **end if**12:    Set the slot index $k\leftarrow n_{b,s}+1$13:    Assign the instance-level constraints to the slot-level variables:14:          $\Delta X_{b,s,k}^{b}\leftarrow \Delta X_{b,i}^{b}$15:          $\Delta Y_{b,s,k}^{b}\leftarrow \Delta Y_{b,i}^{b}$16:          $\Delta \alpha_{b,s,k}^{b}\leftarrow \Delta \alpha_{b,i}^{b}$17:    Construct the target-slot record according to Equation (8):18:          $\tau_{b,s,k}\leftarrow \left(\Delta X_{b,s,k}^{b},\Delta Y_{b,s,k}^{b},\Delta \alpha_{b,s,k}^{b}\right)$19:    Update the required object number $n_{b,s}\leftarrow k$20:    Update the semantic-grouped template entry:21:          $G_{b,s}\leftarrow \left\{n_{b,s},{\left\{\tau_{b,s,j}\right\}}_{j=1}^{n_{b,s}}\right\}$22:**end for**23:Set $G_{b}\leftarrow \left\{G_{b,s}|s\in S_{b}\right\}$24:**return** $G_{b}$

Different from storing translational and angular constraints in two independent lists, the proposed representation stores each target slot as a complete box-local pose tuple. For the *k*-th object instance of semantic category *s* in box type *b*, the target slot is defined as

(8) $$\tau_{b,s,k}=\left(\Delta X_{b,s,k}^{b},\Delta Y_{b,s,k}^{b},\Delta \alpha_{b,s,k}^{b}\right)$$

where $\Delta X_{b,s,k}^{b}$, $\Delta Y_{b,s,k}^{b}$, and  $\Delta \alpha_{b,s,k}^{b}$ denote the box-local relative position and orientation constraints of the object instance with respect to the box frame. Therefore, the position and orientation constraints of the same object instance are kept as a paired slot-level representation.

The output template $G_{b}$ is organized by semantic category as

(9) $$G_{b}=\left\{G_{b,s}|s\in S_{b}\right\}$$

where $S_{b}$ denotes the set of object categories required by box type *b*. For each semantic category *s*, $G_{b,s}$ contains the required object number and the corresponding set of box-local target slots:

(10) $$G_{b,s}=\left\{n_{b,s},{\left\{\tau_{b,s,k}\right\}}_{k=1}^{n_{b,s}}\right\}$$

where $n_{b,s}$ denotes the number of object instances of category *s* required by box type *b*.

#### 3.3.4. Continuous Assignment and State Update Mechanism

For each box type *b*, the semantic-grouped box-centric packing template obtained from Algorithm 2 is represented as $G_{b}=\left\{G_{b,s}|s\in S_{b}\right\}$, where $S_{b}$ denotes the set of object categories required by box type *b*. For each semantic category *s*, $G_{b,s}$ contains the required object number $n_{b,s}$ and the corresponding box-local target slots ${\left\{\tau_{b,s,k}\right\}}_{k=1}^{n_{b,s}}$. Each target slot $\tau_{b,s,k}$ is a complete relative pose tuple defined in Equation (8), rather than separated position and orientation records. This slot-level representation preserves the one-to-one correspondence between the position and orientation constraints of each object instance, which is required for subsequent residual-slot maintenance and target-pose generation.

In execution, the original box-centric packing template $G_{b}$ is used as a fixed reference and is not modified directly. Instead, a residual slot set $R_{b}^{r}$ is maintained in the *r*-th execution round, where $R_{b}^{r}$ stores the target slots extracted from $G_{b}$ that have not yet been assigned or filled. The occupancy state of each slot is defined as

(11) $$\eta_{b,s,k}^{r}=\left\{\begin{array}{cc} 0 & \tau_{b,s,k}\in R_{b}^{r}\\ 1 & \tau_{b,s,k}\notin R_{b}^{r} \end{array}\right.$$

Thus, the state of the continuous execution task is converted from the fixed one-shot packing template $G_{b}$ to a dynamically updated residual slot set $R_{b}^{r}$.

At each execution round, the overhead camera provides the latest scene observation of objects located outside the box regions:

(12) Or=oir=si,Pirbase,αirbasei=1Nr

where $s_{i}$ is the semantic label of object $o_{i}^{r}$, $N_{r}$ is the number of objects observed in this round, and Pirbase and αirbase are its position and rotation angle in the base frame. The pose of each detected box is denoted as

(13) Bb=Pbbase,αbbase=xbbase,ybbase,zbbase,αbbase

For each semantic category *s*, the remaining demand of category *s* for box *b* in the execution round *r* is calculated by

(14) $$q_{b,s}^{r}=\left|\left\{\tau_{b,s,k}|\tau_{b,s,k}\in R_{b}^{r}\right\}\right|$$

During the assignment process, a temporary assignment-level demand ${\hat{q}}_{b,s}^{r}$ is initialized as $q_{b,s}^{r}$. Whenever an object of category *s* is assigned to box *b*, ${\hat{q}}_{b,s}^{r}$ is decreased by one to reserve the corresponding target slot within the current round. Therefore, only boxes with ${\hat{q}}_{b,s}^{r}>0$ are considered valid assignment candidates for subsequent objects of category *s*.

To reduce unnecessary transportation distance while ensuring that each object is used at most once, each currently observed object is assigned to the nearest box that still requires its semantic category. The distance between object $o_{i}^{r}$ and box *b* in the horizontal plane is defined as

(15) d(oir,b)=xirbase−xbbase2+yirbase−ybbase2

The candidate box set for object $o_{i}^{r}$ is defined as

(16) $$B_{s_{i}}^{r}=\left\{b|{\hat{q}}_{b,s_{i}}^{r}>0\right\}$$

If $B_{s_{i}}^{r}$ is not empty, the box assignment is obtained by

(17) $$b_{i}^{*}=\underset{b\in B_{s_{i}}^{r}}{\arg\min}d\left(o_{i}^{r},b\right)$$

Before selecting slots for the assignments in round *r*, the next-round residual slot sets are initialized as $R_{b}^{r+1}\leftarrow R_{b}^{r}$ for all b∈B. For an assigned object $o_{i}^{r}$, the first unoccupied slot $\tau_{b_{i}^{*},s_{i},k_{i}^{*}}$ is selected from the next-round residual slot set $R_{b_{i}^{*}}^{r+1}$. The grasping pose is given directly by the current object pose:

(18) Gir=Pirbase,αirbase

The target placement pose is calculated by transforming the box-centric slot constraint into the base frame. Since the box-local pose constraint is defined in $F_{b}$, while the robot path is generated in $F_{base}$, the rotation angle used for the planar coordinate transformation is

(19) θbi*=π2−αbi*base

The target position is computed as

(20) xg,irbaseyg,irbase=xbi*baseybi*base+cosθbi*−sinθbi*sinθbi*cosθbi*·ΔXbi*,si,ki*bΔYbi*,si,ki*b

and the target height and rotation angle are given by

(21) zg,irbase=αbi*base,αg,irbase=αbi*base−Δαbi*,si,ki*b

Therefore, the executable pick-and-place command generated in round *r* is represented as

(22) Πir=si,Gir,Tir,Tir=xg,irbase,yg,irbase,zg,irbase,αg,irbase

After the assignment result is generated in the current execution round, the residual slot set and the slot occupancy state are updated for the next round. Here, ∖ denotes the set-difference operation, which removes all slots assigned in the current round from the residual slot set:

(23) $$R_{b}^{r+1}=R_{b}^{r}\setminus \left\{\tau_{b_{i}^{*},s_{i},k_{i}^{*}}|\left(o_{i}^{r},b_{i}^{*}\right)\in A^{r},\;b_{i}^{*}=b\right\}$$

For each assigned slot, its occupancy state is updated as

(24) $$\eta_{b_{i}^{*},s_{i},k_{i}^{*}}^{r+1}=1$$

For all other unassigned slots, their occupancy states remain unchanged.

The remaining demand after updating is calculated as

(25) $$q_{b,s}^{r+1}=\left|\left\{\tau_{b,s,k}|\tau_{b,s,k}\in R_{b}^{r+1}\right\}\right|$$

The missing set after round *r* is then calculated as

(26) $$M^{r}=\left\{\left(b,s,q_{b,s}^{r+1}\right)|q_{b,s}^{r+1}>0\right\}$$

which indicates the object categories and quantities that still need to be supplemented.

The redundant object set is defined as

(27) $$E^{r}=\left\{o_{i}^{r}|∄b,\;\left(o_{i}^{r},b\right)\in A^{r}\right\}$$

where $E^{r}$ denotes the redundant object set in round *r*. It contains the observed objects that are not assigned to any box in the current assignment set $A^{r}$. These objects either do not belong to any required semantic category or exceed the remaining demand of their categories.

The whole sorting-and-filling task is completed when

(28) $$\sum_{b\in B}\sum_{s\in S_{b}}q_{b,s}^{r+1}=0$$

If the task is not completed, the updated residual slot sets are preserved, and a new execution round is triggered after the available objects are updated. If the task is completed, the residual slot sets are reinitialized from the target slots contained in the original box-centric packing template $G_{b}$ for the next batch of boxes.

Algorithm 3 summarizes the continuous assignment and state update mechanism. Unlike offline trajectory replay, the proposed mechanism does not assume that all required objects are available before execution or remain unchanged during execution. Instead, it re-observes the available objects outside the box regions at each round, assigns only currently available objects, removes assigned target slots from the residual slot sets, and reports missing or redundant objects. Therefore, external interventions, such as adding missing objects, removing irrelevant objects, or changing object positions, can be handled in the next execution round without re-teaching the packing template.

From the viewpoint of execution feedback, the proposed mechanism forms a task-level closed loop. The system does not simply replay a fixed sequence of demonstrated motions. Instead, after each execution round, the residual slot sets, missing object set, and redundant object set are updated according to the latest observation of available objects. This feedback does not correct the robot trajectory continuously during a single motion primitive, but it corrects the subsequent task allocation and command generation before the next execution round. Thus, execution stability is improved at the task level by preventing already filled slots from being selected again, by avoiding assignment to unavailable objects, and by preserving unfinished target slots until the required objects become available.
**Algorithm 3** Continuous Assignment and State Update for Persistent Sorting-and-Filling  1:**Input:** Box set B, box-centric packing templates $\left\{G_{b}\right\}$, box poses $\left\{B_{b}\right\}$  2:**Output:** Pick-and-place commands $\left\{\Pi_{i}^{r}\right\}$, missing set $M^{r}$, redundant object set $E^{r}$  3:Initialize residual slot sets $R_{b}^{1}\leftarrow \left\{\tau_{b,s,k}|s\in S_{b},\;k=1,\ldots ,n_{b,s}\right\}$ for all b∈B  4:Initialize execution round r←1  5:**while** the system is running **do**  6:    Wait for the execution trigger  7:    **if** all residual slot sets are empty **then**  8:        Reset $R_{b}^{1}\leftarrow \left\{\tau_{b,s,k}|s\in S_{b},\;k=1,\ldots ,n_{b,s}\right\}$ for all b∈B and set r←1  9:    **end if**10:    Acquire the latest scene observation $O^{r}$ of objects located outside the box regions11:    Initialize assignment set $A^{r}\leftarrow ⌀$ and clear the pick-and-place commands for round *r*12:    Initialize next-round residual slot sets $R_{b}^{r+1}\leftarrow R_{b}^{r}$ for all b∈B13:    **for** each box b∈B and each semantic category $s\in S_{b}$ **do**14:        Calculate $q_{b,s}^{r}$ by Equation (14) and set ${\hat{q}}_{b,s}^{r}\leftarrow q_{b,s}^{r}$15:    **end for**16:    **for** each observed object $o_{i}^{r}\in O^{r}$ **do**17:        Obtain its semantic label $s_{i}$ and construct $B_{s_{i}}^{r}=\left\{b|{\hat{q}}_{b,s_{i}}^{r}>0\right\}$18:        **if** $B_{s_{i}}^{r}\neq ⌀$ **then**19:             Assign $o_{i}^{r}$ to $b_{i}^{*}=\arg\min_{b\in B_{s_{i}}^{r}}d\left(o_{i}^{r},b\right)$20:             Add $\left(o_{i}^{r},b_{i}^{*}\right)$ to $A^{r}$ and update ${\hat{q}}_{b_{i}^{*},s_{i}}^{r}\leftarrow {\hat{q}}_{b_{i}^{*},s_{i}}^{r}-1$21:        **end if**22:    **end for**23:    **for** each assignment $\left(o_{i}^{r},b_{i}^{*}\right)\in A^{r}$ **do**24:        Select the first unoccupied slot $\tau_{b_{i}^{*},s_{i},k_{i}^{*}}$ from $R_{b_{i}^{*}}^{r+1}$25:        Calculate the grasping pose Gir=(Pirbase,αirbase)26:        Calculate the target pose $T_{i}^{r}$ by Equations (20) and (21), and generate $\Pi_{i}^{r}=\left(s_{i},G_{i}^{r},T_{i}^{r}\right)$27:        Add the generated command $\Pi_{i}^{r}$ to the pick-place command set of round *r*28:        Update $R_{b_{i}^{*}}^{r+1}=R_{b_{i}^{*}}^{r+1}\setminus \left\{\tau_{b_{i}^{*},s_{i},k_{i}^{*}}\right\}$ and set $\eta_{b_{i}^{*},s_{i},k_{i}^{*}}^{r+1}=1$29:    **end for**30:    **for** each box b∈B and each semantic category $s\in S_{b}$ **do**31:        Calculate the updated remaining demand $q_{b,s}^{r+1}$ from $R_{b}^{r+1}$32:    **end for**33:    Calculate $M^{r}=\left\{\left(b,s,q_{b,s}^{r+1}\right)|q_{b,s}^{r+1}>0\right\}$34:    Calculate $E^{r}=\left\{o_{i}^{r}|∄b,\;\left(o_{i}^{r},b\right)\in A^{r}\right\}$35:    **if** $\sum_{b}\sum_{s}q_{b,s}^{r+1}=0$ **then**36:        Mark the current batch as completed37:    **else**38:        Preserve $\left\{R_{b}^{r+1}\right\}$ and set r←r+139:    **end if**40:**end while**41:**return** $\left\{\Pi_{i}^{r}\right\}$, $M^{r}$, $E^{r}$

## 4. Experiments and Results

**Figure 4:**
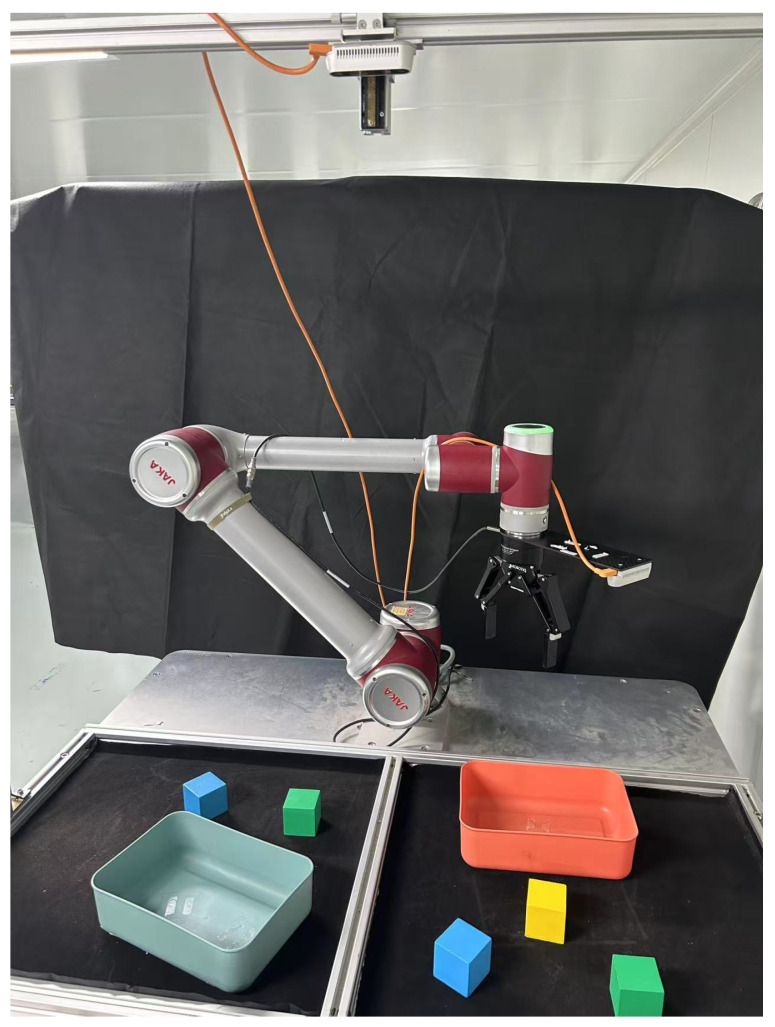
Layouts of the system.

The experimental setup is shown in Figure 4. The workstation has two working areas, each 55 cm in width and 50 cm in height, and is covered with black absorbent cloth. The JAKA Zu7 robot (JAKA Robotics Co., Ltd., Shanghai, China) with a Robotiq two-finger gripper (Robotiq Inc., L’evis, QC, Canada) as an end effector is mounted outside and along the splitting line of the two working areas. The system used two Intel RealSense D435i cameras (Intel Corporation, Santa Clara, CA, USA), serving as the scene camera and the wrist-mounted camera, respectively. The scene camera was suspended above the workbench to provide full coverage of the workspace, while the wrist-mounted camera was installed between the robot end flange and the gripper. In this work, the extrinsic parameters of both the scene camera and the wrist-mounted camera were calibrated using the Park method [31] implemented in the EasyHandEye toolbox (version 0.4.3) [32].

Regarding the bin pick task, for illustration purposes, two types of boxes have been chosen, red and green boxes. All boxes are of the same size, 26 cm in length, 18 cm in width and 8 cm in height. Similarly, there are three types of 5×5 ${\mathrm{cm}}^{2}$ cubes to be picked, namely blue, green, and yellow cubes.

During execution, the robot followed the generated pick-and-place commands using its standard Cartesian motion interface. For each assigned object, the system first moved the manipulator to the grasping pose $G_{i}^{r}$, executed the grasp with the two-finger gripper, and then moved to the target placement pose $T_{i}^{r}$ generated from the box-local slot constraint. The experiments were therefore designed to evaluate the stability of the proposed perception-to-command pipeline and task-level state feedback, rather than to benchmark a new low-level motion controller.

**Figure 5:**
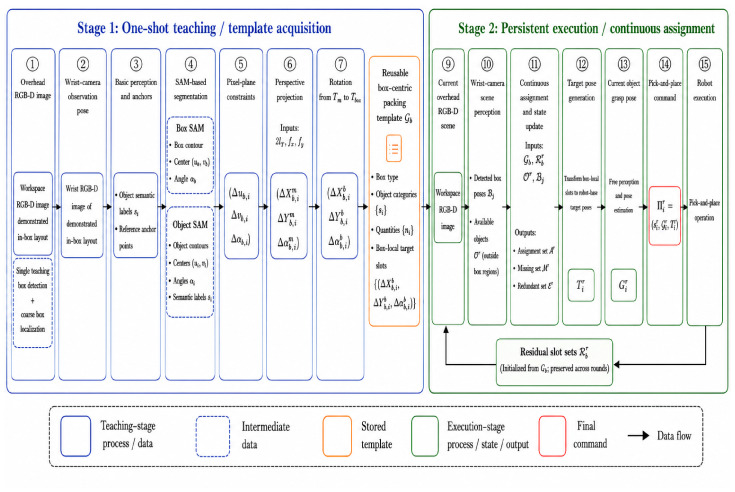
Overall experimental pipeline from raw camera input to final robot execution commands.

To improve the reproducibility of the experiments, the complete perception-to-execution data flow used in the experimental system is summarized in Figure 5. The pipeline starts from raw overhead and wrist-camera RGB-D observations and proceeds through basic perception, SAM-based segmentation, pixel-plane constraint extraction, perspective projection, and the rotation from $F_{m}$ to $F_{box}$ to construct the reusable box-centric packing template $G_{b}$. During execution, the current workspace observation and residual slot sets are used for scene perception, continuous assignment and state update, target pose generation, current object grasp pose estimation, pick-and-place command generation, and final robot execution.

### 4.1. Single-Box Template Transfer Experiment

This experiment was designed to evaluate whether the packing layout taught from a single human demonstration could be recorded as a reusable box-centric template and transferred to a new box pose. The experiment consisted of two stages: one-shot template acquisition and template-based execution. In the first stage, the operator manually placed the required objects inside a target box according to the desired packing layout. The system then observed the demonstrated box, extracted the relative pose constraints of the in-box objects, and stored the semantic-grouped packing template. In the second stage, an empty box of the same type was placed in a different pose, and the robot reproduced the recorded template according to the box pose estimated in the current scene.

The teaching-stage template acquisition procedure used in this experiment corresponds to Stage 1 of Figure 5. During the demonstration, the operator first placed only one target box in the workspace. The vision module was used to recognize the type of box and obtain its pose in the workspace. The wrist-mounted camera was then arranged to observe the demonstrated layout and kept approximately normal to the box plane to capture the demonstrated packaging image. The center and orientation of the box were extracted through SAM-based segmentation. Meanwhile, estimated object centers were used as anchor points for SAM-based object segmentation, from which the relative position constraints of the pixel-plane were obtained. Finally, the pixel-plane constraints were converted into box-local pose constraints in the box coordinate frame, and the semantic-grouped packing template was stored for subsequent execution.

In this experiment, two packing templates corresponding to two different types of boxes were recorded. For each template, the demonstrated packing image was saved as a visual reference to the in-box instruction. Meanwhile, the system extracted a structured box-centric template from the demonstration. The recorded template contains the box type, object semantic categories, object quantities, and the local relative pose constraints of each object with respect to the box frame. Therefore, the template is independent of the global position and orientation of the demonstrated box and can be reused when another box of the same type is detected.

**Figure 6:**
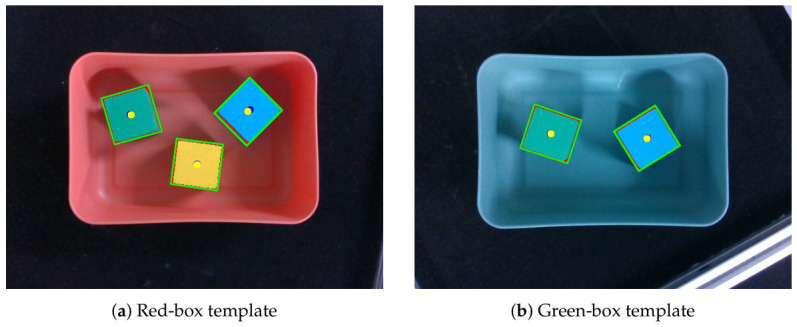
Demonstrated packing layouts and segmentation results for the two recorded templates. The green contours indicate the segmented object boundaries, and the yellow points denote the extracted object centers.

As shown in Figure 6 and Table 1, the proposed method was able to record different packing layouts from demonstrations in one single shot. For the red-box template, three object categories were recorded, including one blue cube, one green cube, and one yellow cube. For the green-box template, two object categories were recorded, including one blue cube and one green cube. For each object instance, the semantic label and the relative pose constraint in the box coordinate frame were stored.

Although the demonstrated templates were obtained from specific box poses, the stored information was represented in the local coordinate frame of the box. This representation allows the same template to be transferred to a new box instance by transforming the local target pose into the robot base frame according to the currently observed box pose. In this way, the human demonstration is converted into an executable and reusable packing template. The robot does not need to replay the demonstrated trajectory or rely on the original global position of the demonstrated box. Instead, it only uses the recorded local relative pose constraints to generate the target placement poses during execution.

After the two templates were recorded, each template was executed 12 times in different box poses. During execution trials, the target box was placed in different positions and orientations in both the left and right working regions. For each trial, the robot generated the target placement poses by transforming the constraints of the recorded box-local pose focused on the box into the robot base frame according to the currently detected box pose.

The final packing performance was evaluated using the in-box placement position error. During template recording, the manually drawn object contours were used as the reference contours. After each placement test, the contour of the placed object was detected again and the center deviation between the contour of the placed object and the reference contour was calculated. For the ith placed object, the position error was defined as

(29) $$e_{\mathrm{place}}^{i}=\sqrt{{\left(x_{i}^{template}-x_{i}^{place}\right)}^{2}+{\left(y_{i}^{template}-y_{i}^{place}\right)}^{2}}$$

where $\left(x_{i}^{template},y_{i}^{template}\right)$ denotes the center of the reference contour in the taught template and $\left(x_{i}^{place},y_{i}^{place}\right)$ denotes the center of the object contour placed after execution. The unit of the placement error is millimeter.

In each execution round, the pick-and-place command $\Pi_{i}^{r}$ and the target placement pose $T_{i}^{r}$ follow the definitions of Equation (22), while the grasp pose $G_{i}^{r}$ is defined in Equation (18). In this experiment, the placement position error was used as the main quantitative evaluation metric because the purpose was to evaluate the transfer accuracy of the recorded box-centric position constraints. The planar target position component of $T_{i}^{r}$ is generated directly from the recorded local position constraints and the currently detected box pose.

To provide a more complete robustness analysis beyond the boxplot visualization, the standard deviation, minimum error, maximum error, and 95% confidence interval of the placement error were further calculated for each object category and for each recorded template. The confidence interval was calculated for the mean placement error using Student’s *t*-distribution. The statistical results are summarized in Table 2.

**Figure 7:**
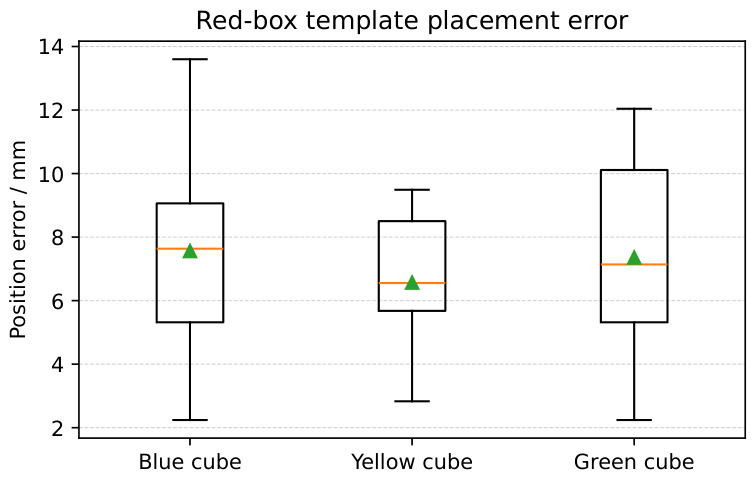
Boxplot of the placement position errors for the red-box template. The orange line and green triangle denote the median and mean values, respectively.

**Figure 8:**
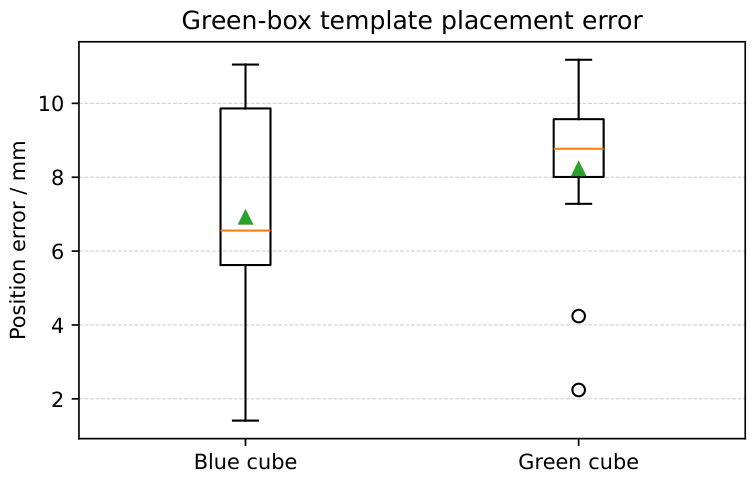
Boxplot of the placement position errors for the green-box template. The orange line, green triangle, and open circles denote the median, mean, and outlier values, respectively.

As shown in Figure 7 and Figure 8 and Table 2, both recorded templates achieved stable placement accuracy across repeated executions. For the red-box template, the overall mean placement error was 7.16 mm, with a standard deviation of 2.72 mm and a 95% confidence interval of [6.24, 8.08] mm. At the object-category level, the mean errors of the blue, yellow, and green cubes were 7.56 mm, 6.56 mm, and 7.36 mm, respectively, and the maximum error among all red-box trials was 13.60 mm.

For the green-box template, the overall mean placement error was 7.57 mm, with a standard deviation of 2.88 mm and a 95% confidence interval of [6.35, 8.79] mm. The mean errors of the blue and green cubes were 6.91 mm and 8.22 mm, respectively, and the maximum error among all green-box trials was 11.18 mm. Although several individual trials showed larger deviations, the confidence intervals of the overall template errors remained within a narrow range around the mean values. This indicates that the recorded box-centric position constraints can be transferred to boxes with different poses with repeatable placement accuracy. The remaining deviations are mainly associated with box-pose estimation uncertainty, gripper–object contact variation during picking, and small object motion before or after placement.

Since the proposed template stores both translational and angular relative pose constraints, the preservation of object orientation after placement was also examined. In principle, the target orientation αg,irbase in $T_{i}^{r}$ is determined by the detected box orientation and the fixed relative orientation constraint stored in the template. After template recording, this relative orientation constraint remains unchanged and is transferred together with the box-local position constraint when generating the placement pose.

**Figure 9:**
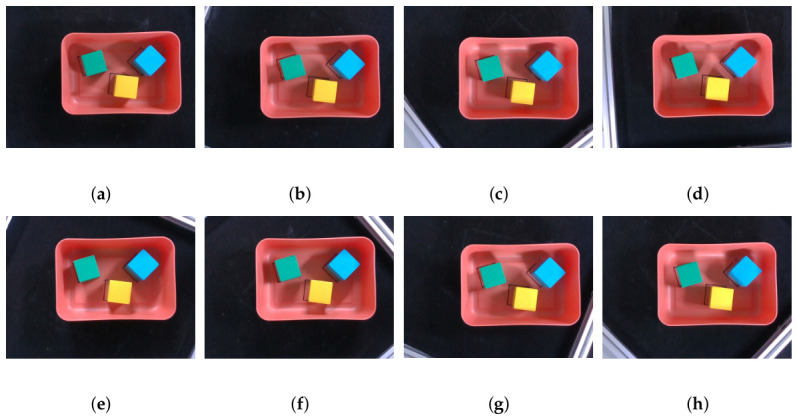
Representative post-execution orientation-preservation examples for the red-box template: (**a**–**h**) correspond to examples 1–8, respectively. In each subfigure, the black template footprints indicate the recorded target orientations, and the placed cubes show the reproduced orientations after execution.

However, a precise numerical orientation error was not reported in this experiment because the available reference marks were manually drawn on the bottom of the box. The visible marker-line thickness, partial occlusion by the placed cubes, perspective effects, and image-resolution limitations make small angular deviations difficult to measure reliably from the post-placement images. Therefore, instead of reporting potentially inaccurate small-angle values, this work provides a visual orientation-preservation assessment based on representative post-execution images. In Figure 9, the black marker contours indicate the target object footprints recorded during template teaching, while the placed cubes indicate the executed object poses. Eight representative trials from the twelve red-box executions are displayed for visual illustration. The red-box template is used only as a representative example; the same box-centric relative-orientation transfer formulation is used for all recorded box types in this experiment.

In addition to the translational error distribution, Figure 9 provides representative visual evidence of orientation preservation. Although the current measurement setup does not support reliable small-angle error quantification, the displayed examples show that the placed cubes maintained orientations visually consistent with the corresponding template footprints. Since all recorded box types in this experiment use the same box-local angular constraint $\Delta \alpha_{b,s,k}^{b}$ and the same orientation-transfer equation in Equation (21), this qualitative observation supports the orientation-preservation capability of the proposed representation beyond the illustrated red-box case.

In general, both templates achieved stable placement performance in 12 trials. The results demonstrate that the recorded box-centric relative pose constraints can be successfully transferred to boxes placed at different positions and orientations. The added statistical indicators, including standard deviation, minimum and maximum errors, and confidence intervals, further provide quantitative evidence for the repeatability of the single-box template transfer experiment. The representative post-execution images further indicate that the angular component of the relative pose constraint can be preserved visually, although the current setup does not provide reliable numerical orientation-error measurement. Therefore, the proposed one-shot template representation is effective in reproducing the learned packing layouts without relying on the original global pose of the demonstrated box.

### 4.2. Multi-Box Sorting-and-Filling Experiment

This experiment was conducted to evaluate whether the proposed continuous assignment and state update mechanism could support multi-box sorting-and-filling under changing object availability. The two recorded templates were used simultaneously in the workspace. As shown in Figure 6, the red-box template required one blue cube, one green cube, and one yellow cube, while the green-box template required one blue cube and one green cube. For clarity, *B*, *G*, and *Y* denote the semantic categories of the blue cube, the green cube, and the yellow cube, respectively. The red box and the green box are denoted as $b_{\mathrm{red}}$ and $b_{\mathrm{green}}$.

At the beginning of each batch, the set of residual slots was initialized from the recorded templates as $R_{b_{\mathrm{red}}}^{1}=\left\{B,G,Y\right\}$ and $R_{b_{\mathrm{green}}}^{1}=\left\{B,G\right\}$. In each execution round, the overhead camera observed the objects outside the box regions and generated the current observation set $O^{r}$. Based on the remaining residual slots, the system generated the assignment set $A^{r}$ and the pick-and-place commands $\Pi_{i}^{r}=\left(s_{i},G_{i}^{r},T_{i}^{r}\right)$, where $G_{i}^{r}$ is the grasp pose, and $T_{i}^{r}$ is the target position pose. After each execution round, the assigned slots were removed from the residual slot sets, while the missing set $M^{r}$ and the redundant object set $E^{r}$ were updated.

**Figure 10:**
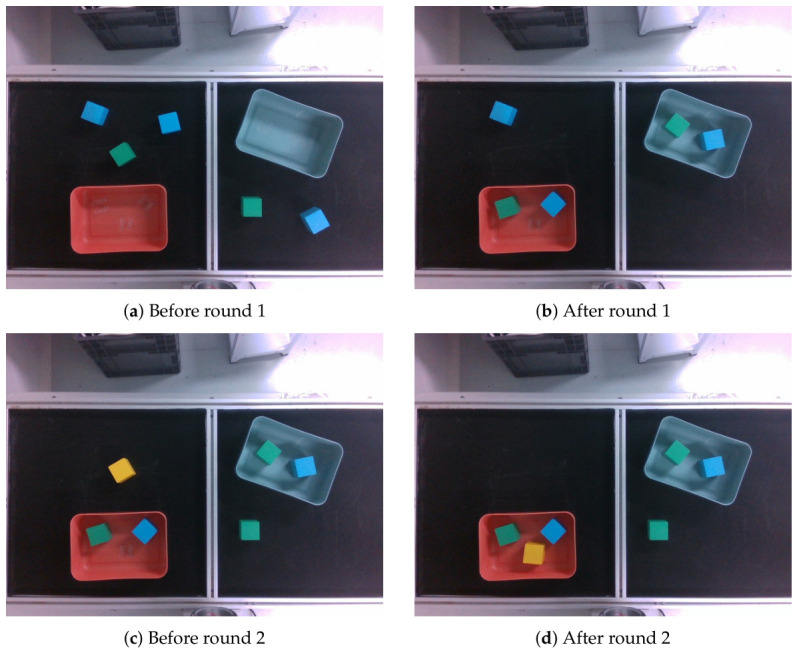
First multi-box sorting-and-filling case. The system preserved the residual slot state after the first round and completed the missing yellow-cube placement in the second round.

As shown in Figure 10 and Table 3, the initial scene contained three blue cubes and two green cubes, but no yellow cube. Based on the residual demands of the two boxes, the system assigned one blue cube and one green cube to each box in the first round. After round 1, the green-box template was completed, while the red box still lacked one yellow cube. Therefore, the residual slot set was updated to $R_{b_{\mathrm{red}}}^{2}=\left\{Y\right\}$ and $R_{b_{\mathrm{green}}}^{2}=⌀$. The missing set was reported as $M^{1}=\left\{\left(b_{\mathrm{red}},Y,1\right)\right\}$.

Before the second round, a yellow cube was added to the workspace. Meanwhile, an additional green cube was present in the scene. Since the only remaining residual slot required a yellow cube for the red box, the system generated a command to place the yellow cube into the red box. The extra green cube was not assigned to any valid box and was therefore included in the set of redundant objects $E^{2}$. After the second round, all residual slot sets were empty, indicating that the current batch was completed.

**Figure 11:**
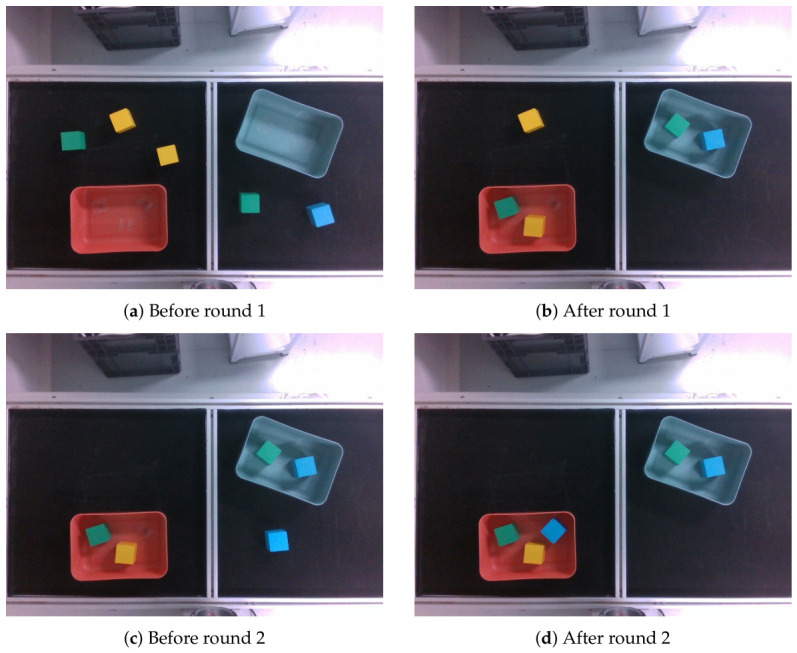
Second multi-box sorting-and-filling case. The system detected the missing blue cube after the first round and completed the remaining residual slot after the scene was updated.

As shown in Figure 11 and Table 4, the second case was initialized with one blue cube, two green cubes, and two yellow cubes. The total demand for the two templates required two blue cubes, two green cubes, and one yellow cube. Therefore, one blue cube was missing and one yellow cube was redundant in the first round. According to the assignment result, the red box received one green cube and one yellow cube, while the green box received one blue cube and one green cube. After the first round, the residual state was preserved as $R_{b_{\mathrm{red}}}^{2}=\left\{B\right\}$, which means that the red box still required one blue cube. The system reported the missing set $M^{1}=\left\{\left(b_{\mathrm{red}},B,1\right)\right\}$ and the redundant object set $E^{1}=\left\{Y\right\}$.

Before the second round, a blue cube was added to the workspace. Since the only remaining residual slot required a blue cube for the red box, the system directly assigned the newly added blue cube to the red box and generated one pick-and-place command. After round 2, both residual slot sets became empty, and the missing and redundant sets were also empty. This shows that the proposed mechanism can preserve the unfinished task state and continue execution after external object supplementation.

The two cases demonstrate the function of the continuous assignment and state update mechanism in multi-box sorting-and-filling tasks. Instead of assuming that all required objects are available before execution, the system updates the residual slot sets after each round and reports the missing and redundant object sets according to the latest scene observation. When missing objects are supplemented later, the system does not need to restart the task or re-record the template. Instead, it uses only the preserved residual slot sets to generate the next-round assignment and pick-and-place commands. Therefore, the proposed method enables persistent execution under dynamic object availability.

These two cases extend the validation from single-box template transfer to simultaneous execution with multiple target boxes. In the current physical workcell, the experiments were limited to two boxes because of the reachable tabletop area and the need to avoid additional inter-box collision constraints. Nevertheless, the assignment and residual-slot update formulation is defined over a general box set B, rather than being restricted to two boxes.

### 4.3. Additional Feasibility Validation with Different Object Shapes and Containers

To further examine the applicability of the proposed framework beyond the original red-box and green-box settings, additional feasibility tests were conducted using two new container types and additional object geometries. The newly introduced containers included a white box and a wood-colored box. The white box had a size of 26cm×18cm×7cm, while the wood-colored box had a size of 31.8cm×24.4cm×10.4cm. In addition to cube objects, two additional object shapes were introduced, including cylinders and a rectangular cuboid. The cylinders had a diameter and height of approximately 5cm×5cm, and the rectangular cuboid had a size of approximately 10cm×5cm×5cm. These tests were designed to verify whether the proposed perception-to-template and template-to-execution pipeline could be extended to different container appearances, container sizes, and object shapes.

**Figure 12:**
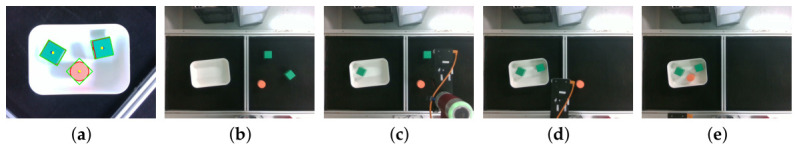
Additional feasibility validation using the white-box template. (**a**) Template acquisition; (**b**) initial scene; (**c**) Step 1; (**d**) Step 2; (**e**) Step 3. In subfigure (**a**), the yellow points indicate the reference anchor points used as SAM prompt points and for center estimation, while the green contours indicate the refined object contours used for box-local pose extraction.

**Figure 13:**
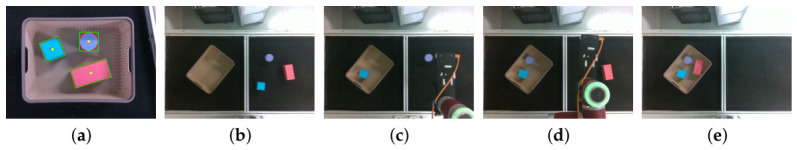
Additional feasibility validation using the white-box template. (**a**) Template acquisition; (**b**) initial scene; (**c**) Step 1; (**d**) Step 2; (**e**) Step 3. In subfigure (**a**), the yellow points indicate the reference anchor points used as SAM prompt points and for center estimation, while the green contours indicate the refined object contours used for box-local pose extraction.

As shown in Figure 12 and Figure 13, the proposed framework was able to acquire templates from containers with different appearances and dimensions and execute the corresponding placement sequence for objects with different shapes. The white-box test included two green cubes and one orange cylinder, while the wood-colored box test included a blue cube, a purple cylinder, and a red rectangular cuboid. These results indicate that the proposed box-centric template representation is not limited to the original cube-only examples. Since the template records semantic categories, quantities, and box-local relative pose constraints, the same processing pipeline can be applied to different container types and object geometries as long as the objects can be recognized by the basic sensing module and refined by the SAM-based segmentation module.

Therefore, this additional validation improves the external validity of the experimental evaluation with respect to object categories, object geometries, and container variations.

### 4.4. Quantitative Baseline and Ablation Study

To address the quantitative baseline comparison and ablation analysis, two experimentally verifiable components were further examined in this subsection: SAM-based contour refinement during template acquisition and residual-slot updating during persistent execution. The SAM ablation compares the previous recognition method [29] with the proposed SAM-refined segmentation under different exposure settings. The residual-slot analysis is discussed based on the two multi-box sorting-and-filling cases, where missing objects are supplemented after the first execution round.

For the segmentation ablation, the template acquisition process was tested under nine exposure settings from 600 to 1400. The suffix numbers 6, 7, …, 14 in the image names correspond to exposure values 600, 700, …, 1400, respectively. The previous method directly used the contour results obtained from the basic sensing and recognition module, while the proposed method used the reference points obtained by the previous method as prompts for SAM refinement. A recognition result was regarded as clean if all three cube contours were extracted without additional false object contours.

**Figure 14:**
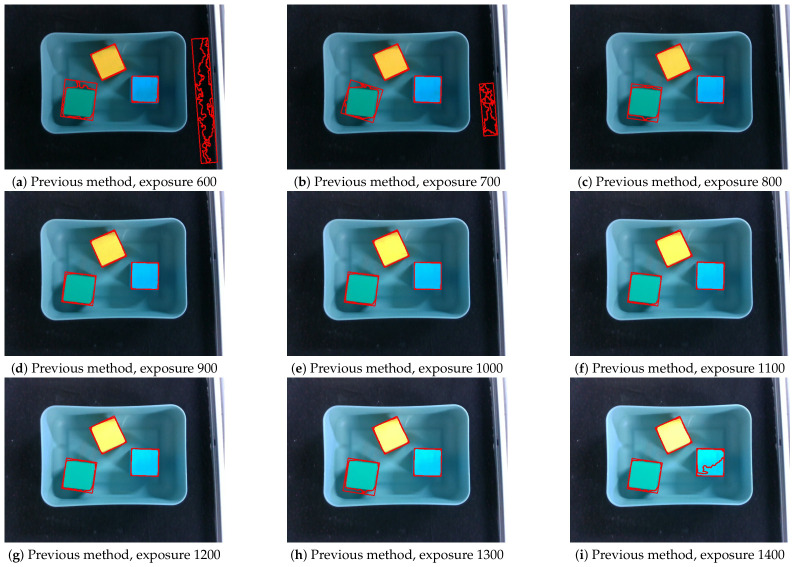
Recognition results obtained by the previous method under different exposure settings. The red contours indicate the object contours extracted by the previous method and used for subsequent reference-point, center, and orientation estimation. Distorted or additional red contours indicate unstable recognition results under the corresponding exposure settings.

**Figure 15:**
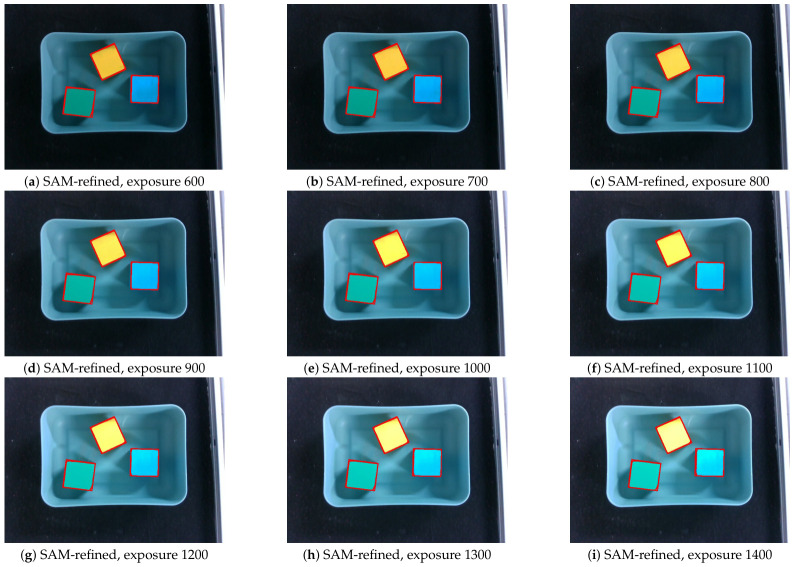
SAM-refined segmentation results under different exposure settings.The red contours indicate the object contours extracted by the previous method and used for subsequent reference-point, center, and orientation estimation. Distorted or additional red contours indicate unstable recognition results under the corresponding exposure settings.

As shown in Figure 14 and Figure 15, the previous method was more sensitive to exposure variation. Under several exposure settings, it produced distorted cube contours or additional false contours outside the target objects. These unstable contours may affect the reference point, object center, and orientation used for template acquisition. By contrast, the SAM-refined method preserved the three cube contours more consistently across the tested exposure range. As summarized in Table 5, the previous method achieved clean recognition in 6 out of 9 exposure settings, whereas the SAM-refined method achieved clean recognition in all 9 exposure settings. This result indicates that SAM refinement improves the robustness of pixel-plane constraint recognition and reduces the dependence on manually tuned color-threshold parameters.

The effect of residual-slot updating was further analyzed based on the two multi-box sorting-and-filling cases shown in Figure 10 and Figure 11. In both cases, one required object was absent in the first execution round and was supplemented before the second round. With residual-slot updating, the system did not restart the complete template after the first round. Instead, it preserved only the unfinished target slots, such as $R_{b_{\mathrm{red}}}^{2}=\left\{Y\right\}$ in the first case and $R_{b_{\mathrm{red}}}^{2}=\left\{B\right\}$ in the second case. Therefore, after the missing object was added, the next-round assignment was generated only for the remaining target slot.

If residual-slot updating is removed, the system degenerates into a static one-round assignment process. Such a baseline can only assign objects that are available in the current observation and cannot explicitly maintain the unfilled slot after the current execution round. When a missing object is supplemented later, the baseline has no residual-slot memory to indicate which specific target slot still needs to be filled. Simply restarting the task is also not equivalent to residual-slot updating, because restarting reinitializes the full template and the system no longer knows which slots have already been occupied in the previous round. As a result, duplicate placement commands may be generated for already filled slots, which can lead to placement conflicts or execution failure. This analysis shows that residual-slot updating is necessary for preserving unfinished task states under external object supplementation, rather than merely re-executing a static assignment process.

### 4.5. Comparison with Related Methods

To further clarify the characteristics of the proposed framework according to the reviewer-suggested comparison dimensions, three closely related methods are selected for comparison: Lu et al. [1], Ding et al. [5], and Du et al. [6]. These methods are related to one-shot visual imitation, template-based task representation, and demonstration-based sorting programming, respectively. Since they were evaluated with different robots, objects, task settings, and metrics, a direct numerical comparison is not fully applicable. Therefore, a qualitative functional comparison is provided in Table 6.

As shown in Table 6, the proposed framework shares the low-demonstration burden with the related methods, but differs in the form of task knowledge extracted from the demonstration. Lu et al. use a one-shot video demonstration to reproduce multi-step pick-and-place operations, while Ding et al. construct reusable manipulation templates for symmetric-object operations. Du et al. record sorting trajectories, object–box correspondences, and key path features from human demonstration. In contrast, the proposed method requires only one in-box layout demonstration and converts the demonstrated arrangement into a box-centric packing template containing semantic categories, object quantities, and slot-level relative pose constraints.

The comparison also shows that the proposed method provides stronger support for constrained sorting-and-filling tasks. The relative pose of each target object is represented as a box-local slot tuple, which enables the taught layout to be transferred to boxes with different global positions and orientations. In addition, the continuous assignment and state update mechanism maintains residual slots, reports missing and redundant objects, and updates object-to-slot assignments across execution rounds. Here, dynamic scene adaptation refers to task-level re-observation and state updating under changes in object availability, rather than low-level visual-servo correction during a single motion primitive. Therefore, compared with the related methods, the proposed framework is more suitable for multi-box and persistent sorting-and-filling tasks in which the target boxes, available objects, and unfinished task states may change during execution.

## 5. Conclusions

This paper proposed a box-centric one-shot teaching framework for persistent robotic sorting-and-filling tasks with relative pose constraints. Instead of recording and replaying a demonstrated trajectory, the proposed method converts a single human-demonstrated in-box layout into a reusable box-centric packing template. The template records object semantic categories, required quantities, and box-local target slots with both translational and angular relative pose constraints. By representing the taught layout in the local coordinate frame of the box, the recorded template can be transferred to boxes with different global positions and orientations.

The proposed framework also integrates reference-prompted SAM-based contour refinement, pixel-plane to box-local pose conversion, and task-level pick-and-place command generation. During execution, a continuous assignment and state updates mechanism maintains residual target slots, updates object-to-slot allocation, and reports missing and redundant objects across successive execution rounds. This allows the system to continue an unfinished sorting-and-filling task after external scene changes without re-teaching the packing template.

Experiments on a robotic packing platform verified the effectiveness of the proposed framework. In the single-box template transfer experiment, two recorded templates were executed under different box poses for 12 trials each, achieving mean placement errors of 7.16mm and 7.57mm, respectively. Representative post-execution images further showed that the relative object orientations were visually preserved with respect to the taught template footprints, supporting the angular component of the recorded relative pose constraints. The multi-box sorting-and-filling experiments demonstrated that the system could preserve unfinished residual slots, detect missing and redundant objects, and continue execution after object supplementation.

Additional feasibility validation further showed that the proposed pipeline could be extended to new container types and object geometries, including white and wood-colored boxes, cylinders, and a rectangular cuboid. The ablation study under nine exposure settings also showed that SAM refinement improved the robustness of template acquisition compared with the previous recognition method. Together with the comparison to related methods, these results indicate that the proposed framework provides explicit box-centric goal representation, semantic quantity recording, relative pose preservation, multi-box filling, and persistent task-level execution.

Future work will extend the framework to more complex and less structured scenarios. Although the revised experiments include additional object categories, different object geometries, different container types, two-box execution, changing object availability, redundant objects, missing-object supplementation, and exposure variation, dense clutter, severe occlusion, and larger-scale multi-container scenes are not fully covered by the current experimental platform. Practical deployment in these more complex scenes requires additional perception robustness, grasp selection, collision-aware motion planning, and recovery mechanisms. Therefore, larger-scale validation involving more boxes, denser scenes, stronger occlusion, and more diverse object categories will be investigated in future work with an expanded workspace and a more complete planning system.

## Figures and Tables

**Table 1:** Recorded box-centric packing templates extracted from one-shot demonstrations.

Box Type	Object Type	Count	$\Delta X_{\mathrm{box}}$ (m)	$\Delta Y_{\mathrm{box}}$ (m)	$\Delta \alpha_{\mathrm{box}}$ (deg)
Red box	Blue cube	1	0.0592	0.0284	44.80
	Green cube	1	−0.0678	0.0252	15.75
	Yellow cube	1	0.0039	−0.0291	−7.63
Green box	Blue cube	1	0.0487	0.0015	31.50
	Green cube	1	−0.0562	0.0070	−18.73

**Table 2:** Placement-error statistics for the single-box template transfer experiment.

Template	Object	*n*	Mean/mm	SD/mm	Min./mm	Max./mm	95% CI/mm
Red box	Blue cube	12	7.56	2.97	2.24	13.60	[5.68, 9.45]
	Yellow cube	12	6.56	2.20	2.83	9.49	[5.17, 7.96]
	Green cube	12	7.36	3.05	2.24	12.04	[5.42, 9.30]
	Overall	36	7.16	2.72	2.24	13.60	[6.24, 8.08]
Green box	Blue cube	12	6.91	3.11	1.41	11.05	[4.94, 8.89]
	Green cube	12	8.22	2.60	2.24	11.18	[6.57, 9.87]
	Overall	24	7.57	2.88	1.41	11.18	[6.35, 8.79]

**Table 3:** Semantic-level state update in the first multi-box sorting-and-filling case.

Round	Observation $O^{r}$	Assignments $A^{r}$	Commands $\Pi_{i}^{r}$	Residual SlotsAfter Update	$M^{r}$	$E^{r}$
r=1	$O^{1}=\{$ B:3,G:2,Y:0 }	$A^{1}=\{$ $\;B\to b_{\mathrm{red}},$ $\;G\to b_{\mathrm{red}},$ $\;B\to b_{\mathrm{green}},$ $\;G\to b_{\mathrm{green}}$ }	Four commands: $\Pi_{1}^{1},\;\Pi_{2}^{1},$ $\Pi_{3}^{1},\;\Pi_{4}^{1}$	$R_{b_{\mathrm{red}}}^{2}=\left\{Y\right\}$; $R_{b_{\mathrm{green}}}^{2}=⌀$	$\left\{\left(b_{\mathrm{red}},Y,1\right)\right\}$	{B}
r=2	$O^{2}=\{$ Y:1,G:1 }	$A^{2}=\{$ $\;Y\to b_{\mathrm{red}}$ }	One command: $\Pi_{1}^{2}$	$R_{b_{\mathrm{red}}}^{3}=⌀$; $R_{b_{\mathrm{green}}}^{3}=⌀$	⌀	{G}

**Table 4:** Semantic-level state update in the second multi-box sorting-and-filling case.

Round	Observation $O^{r}$	Assignments $A^{r}$	Commands $\Pi_{i}^{r}$	Residual Slots After Update	$M^{r}$	$E^{r}$
r=1	$O^{1}=\{$ B:1,G:2,Y:2 }	$A^{1}=\{$ $\;G\to b_{\mathrm{red}},$ $\;Y\to b_{\mathrm{red}},$ $\;B\to b_{\mathrm{green}},$ $\;G\to b_{\mathrm{green}}$ }	Four commands: $\Pi_{1}^{1},\;\Pi_{2}^{1},$ $\Pi_{3}^{1},\;\Pi_{4}^{1}$	$R_{b_{\mathrm{red}}}^{2}=\left\{B\right\}$; $R_{b_{\mathrm{green}}}^{2}=⌀$	$\left\{\left(b_{\mathrm{red}},B,1\right)\right\}$	{Y}
r=2	$O^{2}=\{$ B:1 }	$A^{2}=\{$ $\;B\to b_{\mathrm{red}}$ }	One command: $\Pi_{1}^{2}$	$R_{b_{\mathrm{red}}}^{3}=⌀$; $R_{b_{\mathrm{green}}}^{3}=⌀$	⌀	⌀

**Table 5:** Ablation comparison of the previous method and SAM-refined segmentation during template acquisition.

Method	Exposure Settings	Clean Recognitions	Clean Recognition Rate
Previous method	9	6	66.7%
SAM-refined	9	9	100.0%

**Table 6:** Qualitative functional comparison with closely related methods.

Function	Lu et al. [1]	Ding et al. [5]	Du et al. [6]	Proposed
Demonstration requirement	One-shot video demonstration	Template construction from demonstration	Human demonstration of sorting trajectory	One-shot in-box layout demonstration
Template transfer capability	Partial	Yes	Partial	Yes
Relative pose representation	Partial	Partial	Partial	Yes
Semantic quantity and slot recording	No	No	Partial	Yes
Dynamic scene adaptation	No	No	Partial	Yes
Multi-box support	No	No	Partial	Yes
Persistent task execution	No	No	No	Yes

Note: “Yes” indicates that the function is explicitly represented and supported in the reported framework; “Partial” indicates that a related but narrower capability is available; “No” indicates that the function is not directly addressed in the reported task setting.

## Data Availability

There is no new data created in this study. Data sharing is not available for this paper.

## Abbreviation

The following abbreviation is used in this manuscript:

SAM	Segment Anything Model
